# Confounding by Pre-Morbid Functional Status in Studies of Apparent Sex Differences in Severity and Outcome of Stroke

**DOI:** 10.1161/STROKEAHA.117.018187

**Published:** 2017-08-10

**Authors:** Christel Renoux, Janie Coulombe, Linxin Li, Aravind Ganesh, Louise Silver, Peter M. Rothwell

**Affiliations:** From the Centre for Clinical Epidemiology, Lady Davis Institute for Medical Research, Jewish General Hospital, Montréal, Québec, Canada (C.R., J.C.); Departments of Neurology and Neurosurgery (C.R.) and Epidemiology and Biostatistics (C.R.), McGill University, Montréal, Québec, Canada; and Center for Prevention of Stroke and Dementia, Nuffield Department of Clinical Neurosciences, John Radcliffe Hospital, University of Oxford, United Kingdom (C.R., L.L., A.G., L.S., P.M.R.).

**Keywords:** epidemiology, prognosis, sex, stroke, women

## Abstract

**Background and Purpose—:**

Several studies have reported unexplained worse outcomes after stroke in women but none included the full spectrum of symptomatic ischemic cerebrovascular events while adjusting for prior handicap.

**Methods—:**

Using a prospective population-based incident cohort of all transient ischemic attack/stroke (OXVASC [Oxford Vascular Study]) recruited between April 2002 and March 2014, we compared pre-morbid and post-event modified Rankin Scale score (mRS) in women and men and change in mRS score 1 month, 6 months, 1 year, and 5 years after stroke. Baseline stroke-related neurological impairment was measured with the National Institutes of Health Stroke Scale.

**Results—:**

Among 2553 patients (50.6% women) with a first transient ischemic attack/ischemic stroke, women had a worse handicap 1 month after ischemic stroke (age-adjusted odds ratio for mRS score, 1.35; 95% confidence interval, 1.12–1.63). However, women also had a higher pre-morbid mRS score compared with men (age-adjusted odds ratio, 1.58; 95% confidence interval, 1.36–1.84). There was no difference in stroke severity when adjusting for age and pre-morbid mRS (odds ratio, 1.10; 95% confidence interval, 0.90–1.35) and no difference in the pre-/poststroke change in mRS at 1 month (age-adjusted odds ratio, 1.00; 95% confidence interval, 0.82–1.21), 6 months, 1 year, and 5 years. Women had a lower mortality rate, and there was no sex difference in risk of recurrent stroke.

**Conclusions—:**

We found no evidence of a worse outcome of stroke in women when adjusting for age and pre-morbid mRS. Failure to account for sex differences in pre-morbid handicap could explain contradictory findings in previous studies. Properties of the mRS may also contribute to these inconsistencies.

## Abstract 2

Supplemental Digital Content is available in the text.

Many studies have reported greater stroke severity and worse outcome in women albeit with some conflicting results.^1,2^ Potential explanations for these inconsistencies include different source populations, lack of statistical power, and methodological limitations. First, age difference at the time of stroke, if not properly accounted for, may lead to finding a spurious worse outcome in women.^3,4^ Second, another potential confounding factor often overlooked is functional status before the index stroke, which may influence stroke severity and prognosis even after adjustment for age. Disentangling the respective effect of sex, age, and prior handicap is important because it would shift the focus from sex differences to age-related differences in outcomes. In this respect, the properties of the modified Rankin Scale (mRS), the commonly used measure of handicap in stroke research, may also impact the observed sex differences.

Finally, most previous studies did not include the full spectrum of symptomatic ischemic cerebrovascular events, particularly transient ischemic attack (TIA) and minor stroke and also pre-hospital deaths and severe strokes in community institutions. Only by including the full range of event severity can analyses reliably address sex differences in severity outcomes. To our knowledge, no published study has met these 3 conditions. Our objective was, therefore, to assess potential sex differences in the severity and prognosis of ischemic stroke/TIA between men and women in a large prospective population-based study of incidence and outcome of TIA and stroke (OXVASC [Oxford Vascular Study]) while carefully taking age and pre-morbid functional status into account.

## Methods

### Study Design and Source Population

The OXVASC is an ongoing population-based study of the incidence and outcome of all acute vascular events.^5^ The study population comprises 92 728 individuals, irrespective of age, who are registered with ≈100 general practitioners (GPs) in 9 general practices in Oxfordshire, United Kingdom. In the United Kingdom, the vast majority of individuals register with a GP who is the gatekeeper of the health system. The GP provides their primary healthcare and holds a lifelong record of their medical history. All participating practices on OXVASC held accurate age sex patient registers and allowed regular searches of their computerized diagnostic coding systems. All practices refer patients to only 1 secondary care centre.

The OXVASC was approved by the local research ethics committee (OREC A: 05/Q1604/70). All eligible patients gave informed consent, or study assent was obtained from next of kin if the patient was unable to consent.

From the OXVASC source population, we studied all consecutive patients aged ≥18 years with a first TIA or ischemic stroke between April 1, 2002, and March 31, 2014. Cohort entry was taken as the date of the first recorded TIA or stroke occurring during the study period. Patients were followed until death or the end of the study period (September 30, 2014), whichever occurred first.

### Procedures and Data Collection

The OXVASC study has been described in detail elsewhere.^5^ Briefly, capture and near-complete ascertainment of all incident or recurrent vascular events were achieved by means of several overlapping methods of hot and cold pursuit. These included (1) a daily, rapid-access TIA and stroke clinic to which participating GPs and the local emergency department team referred individuals with suspected TIA or minor stroke; (2) daily searches of admissions to medical, stroke, neurology, and other relevant wards; (3) daily searches of the local emergency department attendance register; (4) daily searches of in-hospital death records via the bereavement office; (5) monthly searches of all death certificates and coroner’s reports for out-of-hospital deaths; (6) monthly searches of GP diagnostic coding and hospital discharge codes; and (7) monthly searches of all brain and vascular imaging referrals.

Patients were assessed by a study physician as soon as possible after a cerebrovascular event. A standardized detailed past medical history was recorded, as well as clinical history of the current medical event, findings of the physical examination, current medications, and investigations. All information was recorded from the patients, relatives, their hospital records, and their general practice records. All interventions occurring subsequent to the event were also recorded. If a patient died before assessment, an eyewitness account was obtained whenever possible and any relevant records reviewed. If death occurred outside the hospital or before the investigation, any autopsy result was reviewed. Clinical details were sought from primary care physicians or other clinicians on all deaths of possible vascular pathogenesis.

Handicap was assessed with the mRS,^6^ including pre-morbid mRS before the vascular event of interest. Raters were all trained in the use of the mRS using an instructional DVD with accompanying written materials produced by the University of Glasgow that has been previously used in large-scale clinical trials.^7^ Neurological impairment was assessed as soon as possible after the event with the National Institutes of Health Stroke Scale (NIHSS).^8^ All cases were reviewed by a senior neurologist (P.M.R.), and the presumed pathogenesis of stroke was classified according to the modified Trial of ORG 10172 in Acute Stroke Treatment criteria.^9^

All patients were followed up by a research nurse or study physician at 1, 3, 6, 12, 60, and 120 months from the time of the first TIA or stroke. If a recurrent vascular event was suspected, the patient was assessed by a study neurologist, with recurrent events also identified by the ongoing daily study surveillance.

### Data Analysis

Descriptive statistics were used to summarize the baseline characteristics of the cohort stratified by sex. Ordinal regression analysis was used to compare the handicap (pre-morbid mRS) before the occurrence of the cerebrovascular event, between women and men adjusting for age, history of stroke or dementia, and other variables listed in the Table. Similarly, we compared stroke severity (using NIHSS in 7 categories) between men and women while adjusting for the same variables, as well as for pre-morbid mRS. Handicap after stroke was evaluated in several ways. We first compared mRS scores 1 month, 6 months, 1 year, and 5 years after ischemic stroke between women and men using ordinal regression. Then, to account for pre-morbid handicap in the assessment of poststroke mRS, we assessed change in mRS score between pre-morbid score and 1 month, 6 months, 1 year, and 5 years after stroke using ordinal regression analysis adjusting for age. Change in mRS score was classified in the following 3 categories: no increase, 1 point increase, ≥2 points increase. In a subsequent analysis, we also assessed changes in mRS as a binary outcome (increase, yes/no) using logistic regression. Finally, among patients with a pre-morbid mRS score of ≤2, we compared the percentage of women and men reaching an mRS score of ≥3 at 1 month. Crude and age-adjusted rates of recurrent stroke and all-cause mortality along with 95% confidence intervals (CIs) were estimated, stratified by sex, based on the Poisson distribution. Cox proportional hazards models were used to estimate separately the hazard ratio (HR) of recurrent stroke and all-cause mortality in women compared with men, adjusting for baseline characteristics listed in the Table, as well as for stroke severity. When studying recurrent stroke, the analysis was repeated using Cox proportional hazards models modified for competing risks of death.^10^ All potential confounders were measured at cohort entry. To assess the robustness of our results, all analyses were repeated among patients with an incident (first in life-time rather than just first in study period) cerebrovascular event only and with censoring at the time of any recurrent stroke.

**Table.**
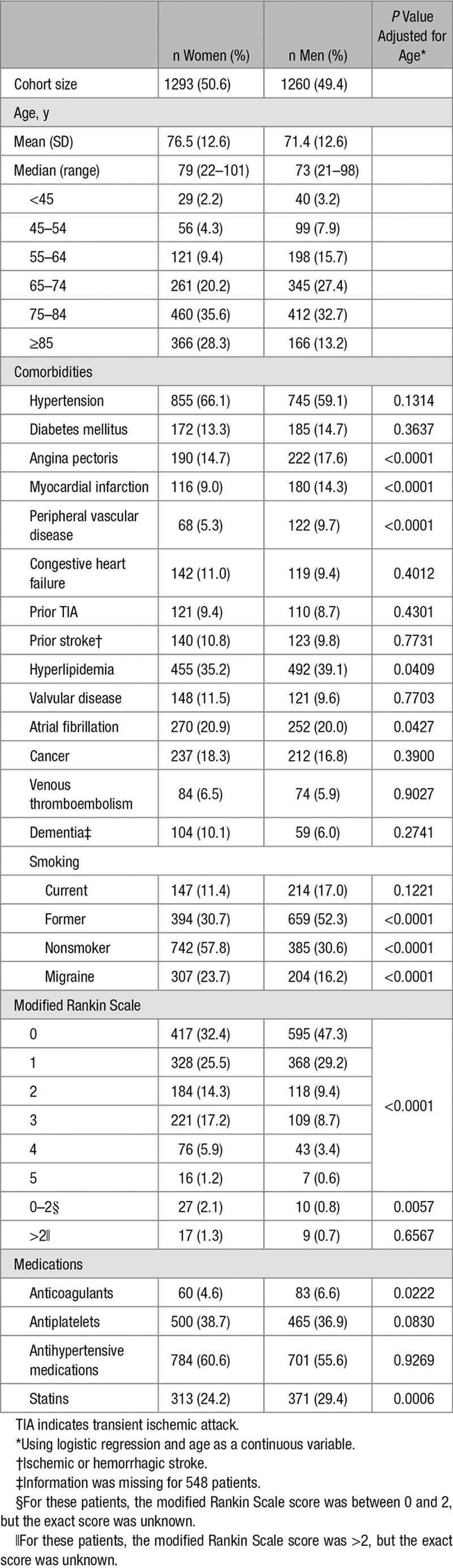
Characteristics of the Cohort of 2553 Patients With a First Ischemic Stroke or Transient Ischemic Attack in the Study Period

CIs were calculated using a significance level of 5%. All statistical procedures were performed using SAS version 9.4 (SAS Institute Inc, Cary, NC).

## Results

**Figure 1.**
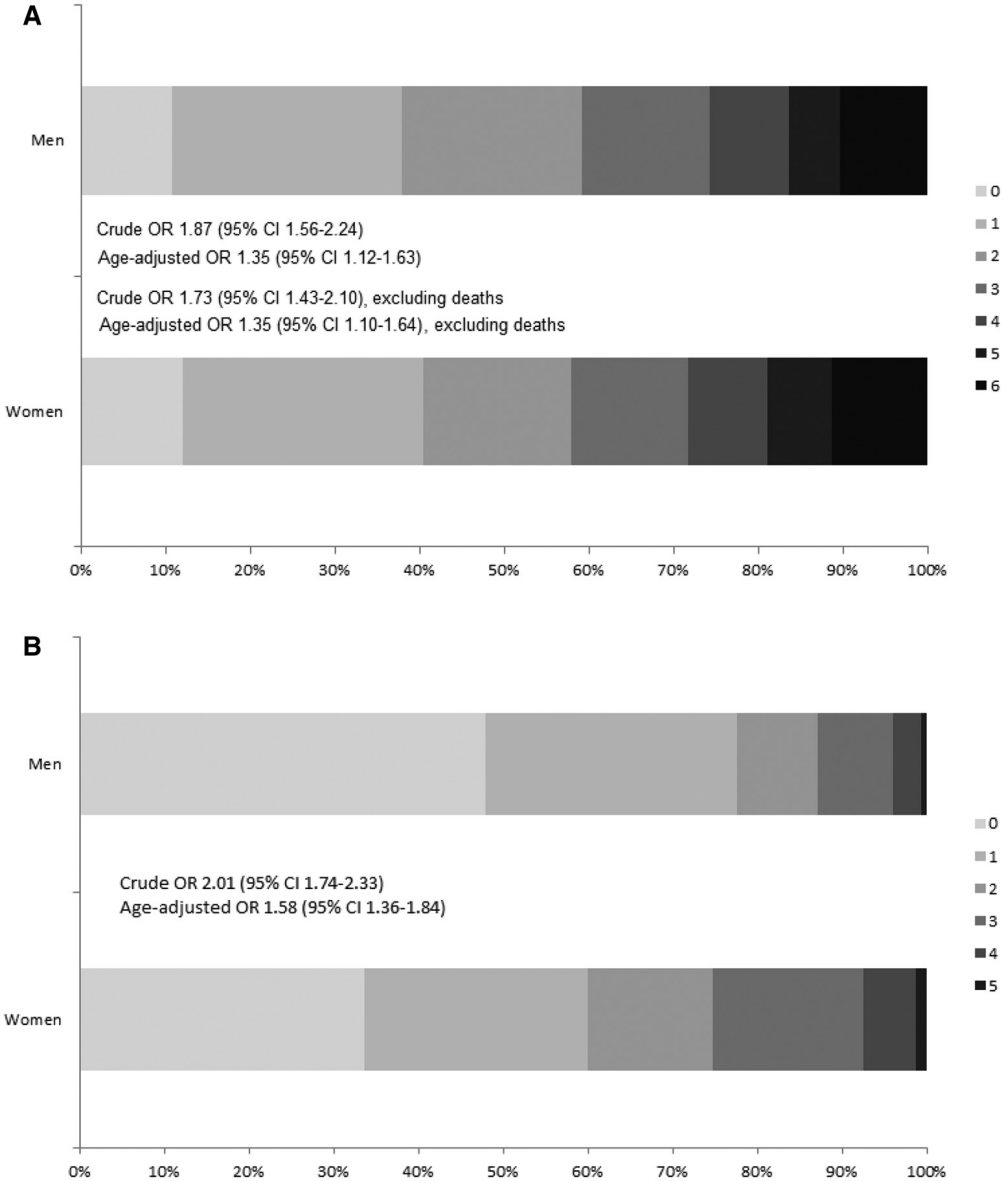
Distribution of modified Rankin Scale (mRS) 1 month after stroke among men and women (**A**) and mRS before stroke (**B**). CI indicates confidence interval; and OR, odds ratio.

The cohort comprised 2553 patients, 1293 (50.6%) women and 1260 (49.4%) men with a first TIA or ischemic stroke during the study period, with a mean follow-up of 4.2 years (SD, 3.4). The baseline characteristics of women and men at the time of the event are shown in the Table. Women were older and had a lower prevalence of coronary artery disease, peripheral vascular disease, and hyperlipidemia compared with men after taking age into account. The same pattern was found in analysis confined to the elderly (≥85 year old, who were predominantly women; Table I in the online-only Data Supplement). However, despite a lesser burden of vascular comorbidities overall, women had a higher pre-morbid mRS score compared with men (ordinal regression odds ratio [OR], 2.01; 95% CI, 1.74–2.33; Figure 1). This difference in pre-morbid mRS persisted after adjusting for age (OR, 1.58; 95% CI, 1.36–1.84), for age, prior stroke, and dementia (OR, 1.62; 95% CI, 1.37–1.92) or for all variables listed in the Table (OR, 1.77; 95% CI, 1.48–2.13). Among 2194 patients with a known marital status, 44.2% of women and 73.2% of men were married or leaving with a partner (age-adjusted OR, 0.34; 95% CI, 0.28–0.41).

A total of 949 (37.2%) patients had a TIA and 1604 (62.8%) had an ischemic stroke with no difference in proportion between women and men (age-adjusted *P*=0.13; Figure I in the online-only Data Supplement). The event was incident in 86.4% of patients and recurrent in 13.6%, with similar figures in both sexes (age-adjusted *P*=0.97). Women had slightly more events originating from the carotid territory than men (74% and 69.7%, respectively) but not after accounting for age (age-adjusted *P*=0.17; Figure II in the online-only Data Supplement). The pathogenesis was most frequently undetermined in both women and men (31.6% and 31.9%, respectively, age-adjusted *P*=0.04) followed by cardioembolic (Figure III in the online-only Data Supplement). The higher frequency of cardioembolic strokes in women (28.1%) compared with men (24.3%) was no longer apparent when taking age into account (age-adjusted *P*=0.97).

**Figure 2.**
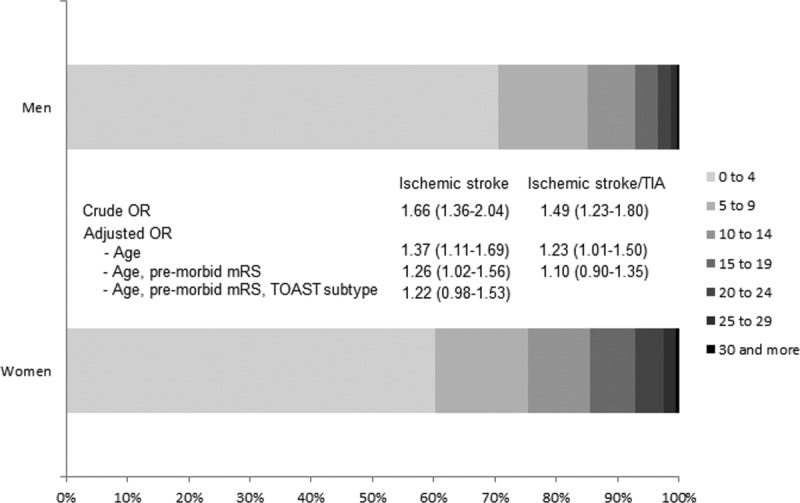
Distribution of National Institutes of Health Stroke Scale score among men and women with ischemic stroke only. mRS indicates modified Rankin Scale; OR, odds ratio; TIA, transient ischemic attack; and TOAST, Trial of ORG 10172 in Acute Stroke Treatment.

Regarding the distribution of cerebrovascular event severity measured by NIHSS, women had more severe events than men (OR, 1.49; 95% CI, 1.23–1.80). However, this difference was halved when adjusted for age (OR, 1.23; 95% CI, 1.01–1.50) and was no longer apparent when adjusting for age and pre-morbid mRS (OR, 1.10; 95% CI, 0.90–1.35), or age, pre-morbid mRS, and other variables listed in the Table (OR, 1.19; 95% CI, 0.94–1.52). The apparent effect of female sex on severity did not vary according to age (*P*=0.85 for interaction). Interestingly, adjusting for all baseline comorbidities listed in the Table but omitting pre-morbid mRS in the model still resulted in an apparently higher severity in women (OR, 1.27; 95% CI, 1.00–1.62). In a subgroup analysis restricted to patients with ischemic stroke only (ie, excluding TIA), women had more severe stroke than men however (age- and mRS-adjusted OR, 1.26; 95% CI, 1.02–1.56; Figure 2). This increased severity was not statistically significant when also adjusting for ischemic stroke subtype (OR, 1.22; 95% CI, 0.98–1.53). Only 21 patients were treated with tissue-type plasminogen activator at the time of stroke, 8 (1%) of 802 women and 13 (1.6%) of 798 men with an ischemic stroke.

**Figure 3.**
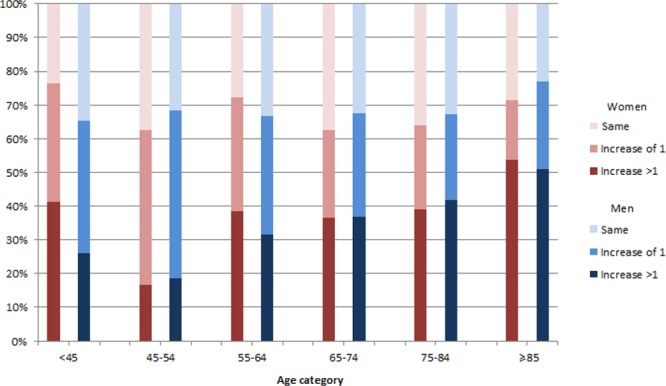
Distribution of the change in modified Rankin Scale score before and 1 month after the ischemic stroke by sex and age category.

Women had a worse mRS score compared with men 1 month after the ischemic stroke, excluding patients with a TIA (crude OR, 1.87; 95% CI, 1.56–2.24 and age-adjusted OR, 1.35; 95% CI, 1.12–1.63; Figure 1). Changes in mRS score from pre-morbid state to 1 month after the ischemic stroke, stratified by age and sex, are shown in Figure 3. Female sex was not associated with a higher risk of increased mRS score either in the whole cohort (age-adjusted OR, 1.00; 95% CI, 0.82–1.21) or in the subgroup of patients aged ≥65 (age-adjusted OR, 0.95; 95% CI, 0.76–1.18). Results were unchanged when assessing mRS change as a binary outcome (age-adjusted OR, 0.91 [95% CI, 0.73–1.14] and 0.86 [95% CI, 0.67–1.10], respectively). Similarly, among patients with a low pre-morbid mRS score (0–2), women were not more likely than men to have a score of ≥3 1 month after the stroke (age-adjusted OR, 1.15; 95% CI, 0.90–1.48). Results were similar for mRS changes at 6 months, except a slightly higher mRS change in women with low pre-morbid mRS (Results I in the online-only Data Supplement). Results were also virtually the same at 1 and 5 years, showing no higher risk of increased mRS score in women compared with men (data not shown). Of note, post-stroke inpatient rehabilitation admission was similar for women and men (43.9% and 41.2%, respectively); this was confirmed after adjusting for age and NIHSS (adjusted OR, 0.91; 95% CI, 0.73–1.13). Poststroke depression was also as frequent in women and men (23.8% and 21.4%, respectively).

During 10 670 person-years of follow-up, 219 (16.9%) women and 199 (15.8%) men had a recurrent ischemic stroke with similar age-adjusted rates, 4.60 (4.01–5.27) per 100 persons per year in women and 4.64 (4.03–5.33) per 100 persons per year in men, respectively. In Cox multivariate analysis, female sex was not associated with a higher risk of recurrent ischemic stroke compared with male (HR, 0.97; 95% CI, 0.79–1.20). Results were unchanged when controlling for competing risks (data not shown).

**Figure 4.**
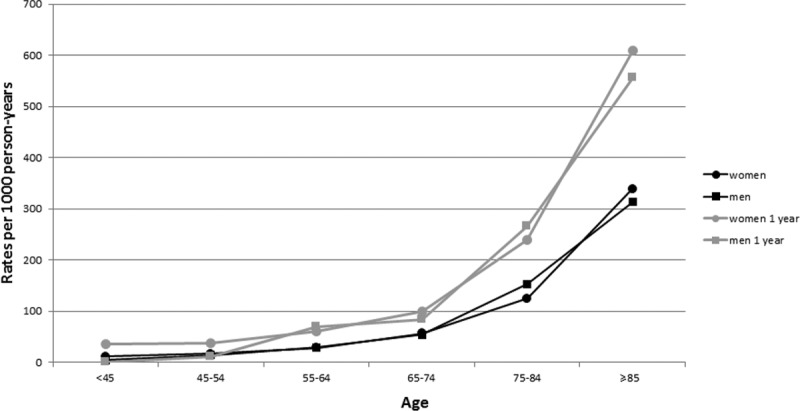
Mortality rates per 1000 person-years during the first year or during the entire study period.

Death occurred in 620 (48%) women and 482 (38%) men during follow-up. Mortality rates increased with age and were similar in women and men when stratified by age whether during the first year or during the entire study period (Figure 4). In Cox univariate analysis, women had a 32% higher risk of death compared with men (HR, 1.32; 95% CI, 1.17–1.48). However, this apparent higher mortality in women was no longer seen when controlling for age (HR, 0.95; 95% CI, 0.84–1.07) and for baseline comorbidities and severity of stroke (HR, 0.82; 95% CI, 0.72–0.94). All results were virtually the same in the sensitivity analyses restricting the cohort to incident cerebrovascular events only (data not shown).

## Discussion

In this population-based cohort study, we showed the impact of potential confounders on the apparent sex differences in severity and outcome of stroke/TIA. Overall, about one third of women and men presented with a TIA, they had a similar proportion of events originating from the anterior circulation, and the cardioembolic pathogenesis was equally represented in men and women when taking age into account. However, women had a higher pre-morbid mRS score compared with men, even when adjusting for age and comorbidities so that the apparently higher severity of cerebrovascular events in women did not remain after adjustment for age and pre-morbid mRS. When ignoring pre-morbid mRS score, women had a worse functional status than men after ischemic stroke. However, change in mRS score 1 month after ischemic stroke was similar between women and men, including among those with low pre-morbid mRS. Finally, women were not at higher risk of recurrent ischemic stroke and had, in fact, a significantly lower mortality rate than men after accounting for age and comorbidities.

Our study included TIAs along with ischemic strokes to cover the whole spectrum of ischemic cerebrovascular events as opposed to most previous studies.^2^ If there was a sex difference in cerebral susceptibility to ischemia, as some have argued, then the proportion of TIA versus stroke might differ. However, the proportion of strokes was similar in both sexes even though women were on average older than men. Moreover, the apparent higher severity of stroke in women was mostly explained by an older age and a worse handicap before the event rather than by sex. Interestingly, the pre-morbid mRS score was still worse in women compared with men after adjusting for age and several comorbidities at baseline. Frailty may still play a role in the sex difference in pre-morbid mRS because of unmeasured confounders. Other factors related to the properties of the mRS scale might also contribute to this difference. Prior studies on stroke severity yielded inconsistent results,^2,11–20^ but they did not account for both age and some measure of handicap before stroke or specifically assess ischemic stroke/TIA while taking both factors into account. Therefore, future studies on sex differences, including clinical trials planning sex-stratified analyses, should properly account for age and collect information on handicap before the event of interest. Moreover, in many instances, it may be more relevant to examine effectiveness based on prior functional status rather than sex because we showed no clinically substantial differences between sexes. In the subgroup of patients with ischemic stroke only, however, women had slightly higher NIHSS scores than men even after taking age and pre-morbid mRS into account because of the greater proportion of cardioembolic stroke in women due to their older age. Of note, as previously reported, the proportion of cardioembolic stroke was not more frequent in women after adjusting for age^21^; previous studies using Trial of ORG 10172 in Acute Stroke Treatment classification and reporting a higher proportion of this subtype in women did not take age into account.^17,22^

Interestingly, change in mRS score between pre-morbid estimate and 1 and 6 months after stroke was similar in women and men. Moreover, rates of recurrence of stroke/TIA were similar, and mortality rate was lower in women in accordance with other studies.^11,23,24^ Conversely, using various scales, some studies found that women had a worse functional status than men after stroke but did not adjust for prior functional status.^2,25^ This finding can be expected if women have already a worse functional status before stroke, as previously reported.^26–28^ One study did not find any difference in poststroke functional status using Barthel Index at 6 months after adjusting for pre-stroke disability and comorbidities,^28^ whereas another still found a worse functional status at 3 months in women in multivariate analysis adjusting for pre-morbid mRS but methodological differences may explain this result.^26^ A recent study on functional outcome after intracerebral hemorrhage also did not show any sex difference in adjusted analyses, including age and pre-morbid mRS among other factors.^29^ Finally, it remains possible that inconsistencies between studies are partly related to the lack of precision of the mRS scale, and our conclusions may not apply to other disability or handicap scales.

Our study has several strengths. OXVASC is a prospective population-based cohort study with nearly complete ascertainment of vascular events in a well-defined geographic region. Therefore, selection bias toward the exclusion of old women with severe stroke living in an institution for instance is unlikely to have occurred. Patients with minor events were also included because the study population was not limited to patients seen in emergency department or hospitalized contrary to many previous studies. Finally, the prospective and standardized data collection by stroke specialists and access to lifelong primary care records ensure completeness and accuracy of data and limit the potential for recall bias. Some potential limitations of our study must, however, be acknowledged. First, the OXVASC population is mostly composed of white patients, and our results may not be entirely generalizable to non-white populations. Second, some residual confounding by unmeasured factors cannot be excluded, but accounting for age and pre-morbid mRS score already corrected fully for apparent sex differences. Moreover, there was no apparent difference in acute care as measured by tissue-type plasminogen activator use and admission to inpatient rehabilitation. Third, although pre-morbid and post-event mRS were measured using standardized forms, scoring was obviously not done while blinded for sex. However, data in OXVASC were not collected for this particular study to test the hypothesis of sex differences in stroke outcomes so that systematic differences in rating are unlikely to entirely explain our results. Similarly, in none of the previous studies evaluating stroke outcomes in men and women was handicap assessed while blinded for sex. Finally, the limitations of using ordinal regression to model mRS scores after stroke are well recognized. However, this approach is currently the most commonly used and allowed us to compare our findings with previous studies.

In conclusion, we found no substantial sex differences in severity, outcome, or recurrence of stroke/TIA. Future studies addressing sex differences in cerebrovascular diseases should take into account functional status before the event of interest as most apparent differences between women and men may be because of age and pre-morbid functional status rather than sex per se.

## Acknowledgments

We are grateful to all the staff in the general practices that collaborated in the Oxford Vascular Study: Abingdon Surgery, Stert St, Abingdon; Malthouse Surgery, Abingdon; Marcham Road Family Health Centre, Abingdon; The Health Centre, Berinsfield; Key Medical Practice; Kidlington; 19 Beaumont St, Oxford; East Oxford Health Centre, Oxford; Church Street Practice, Wantage. We also acknowledge the use of the facilities of the Acute Vascular Imaging Centre, Oxford.

## Sources of Funding

The Oxford Vascular Study is funded by the Wellcome Trust, Wolfson Foundation, and the National Institute for Health Research (NIHR) Oxford Biomedical Research Centre. Dr Renoux is the recipient of a Chercheur-Boursier Award from the Fonds de la recherche du Québec – santé (FRQ-S). Dr Rothwell is in receipt of an NIHR Senior Investigator Award and a Wellcome Trust Senior Investigator Award.

## Disclosures

None.

## Supplementary Material
