# Supplementary material for: Confounding by Pre-Morbid Functional Status in Studies of Apparent Sex Differences in Severity and Outcome of Stroke
Source: Stroke. 2017 Aug 10;48(10):2731–8. doi: 10.1161/STROKEAHA.117.018187 (PMC5610564; doi:10.1161/STROKEAHA.117.018187)
Supplement: Supplementary file 1 [file str-48-2731-s001.pdf]

Confounding by pre-morbid functional status in studies of apparent sex differences in severity and outcome of stroke

ONLINE SUPPLEMENT

Table I. Characteristics of the 532 patients aged 85 years or more with a first ischemic stroke or transient ischemic attack in the study period

Figure I. Distribution of stroke/TIA among men and women by age category

Figure II. Distribution of vascular territory taking imaging into account by sex and age category

Figure III. Distribution of TOAST classification by age and sex

Results I. mRS scores 6 months following the ischemic stroke and change in mRS score from premorbid mRS to 6 months after ischemic stroke in women compared with men

Table I. Characteristics of the 532 patients aged 85 years or more with an ischemic stroke or transient ischemic attack

	N women (%)	*N men (%)*	*P-value adjusted for age**
Cohort size	366 (68.8)	166 (31.2)	
Age			
Mean (SD)	89.5 (3.6)	88.1 (2.9)	
Median (range)	89 (85-101)	87 (85-98)	
85-89	199 (54.37)	123 (74.10)	
≥90	167 (45.63)	43 (25.90)	
Comorbidities			
Hypertension	252 (68.85)	103 (62.05)	0.0693
Diabetes	34 (9.29)	15 (9.04)	0.7965
Angina pectoris	80 (21.86)	40 (24.10)	0.5005
Myocardial infarction	46 (12.57)	30 (18.07)	0.1662
Peripheral vascular disease	27 (7.38)	15 (9.04)	0.7908
Congestive heart failure	67 (18.31)	31 (18.67)	0.7862
Prior TIA	37 (10.11)	22 (13.25)	0.2693
Prior stroke[†]	40 (10.93)	27 (16.27)	0.1438
Hyperlipidemia	95 (25.96)	46 (27.71)	0.7114
Valvular disease	60 (16.44)	21 (12.80)	0.2428
Atrial fibrillation	122 (33.33)	52 (31.33)	0.8420
Cancer	87 (23.77)	41 (24.70)	0.7985
Venous thromboembolism	26 (7.10)	11 (6.63)	0.9217
Dementia[‡]	51 (17.83)	17 (13.08)	0.2261
Smoking			
Current	10 (2.77)	3 (1.81)	0.4508
Former	106 (29.44)	111 (66.87)	**<0.0001**
Non-smoker	243 (67.50)	52 (31.33)	**<0.0001**
Migraine	57 (15.57)	22 (13.25)	0.3488
Modified Rankin scale			
0	57 (15.83)	31 (18.79)	
1	62 (17.22)	48 (29.09)	**0.0316**
2	70 (19.44)	32 (19.39)	

	N women (%)	*N men (%)*	*P-value adjusted for age**
3	110 (30.56)	37 (22.42)	
4	40 (11.11)	12 (7.27)	
5	8 (2.22)	0 (0.00)	
0-2[§]	3 (0.83)	1 (0.61)	0.8413
>2[ǀ]	10 (2.78)	4 (2.42)	0.9465
Medications			
Anticoagulants	14 (3.83)	11 (6.63)	0.1700
Antiplatelets	178 (48.63)	77 (46.39)	0.4756
Antihypertensive medications	236 (64.48)	101 (60.84)	0.2235
Statins	66 (18.03)	35 (21.08)	0.9459

Abbreviations: SD standard deviation. TIA transient ischemic attack

*Using logistic regression and age as a continuous variable

[†]ischemic or hemorrhagic stroke

[‡]Information was missing for 7 patients

[§]For these patients the modified Rankin scale was between 0 and 2 but the exact score was unknown

[ǀ]For these patients the modified Rankin scale was greater than 2 but the exact score was unknown

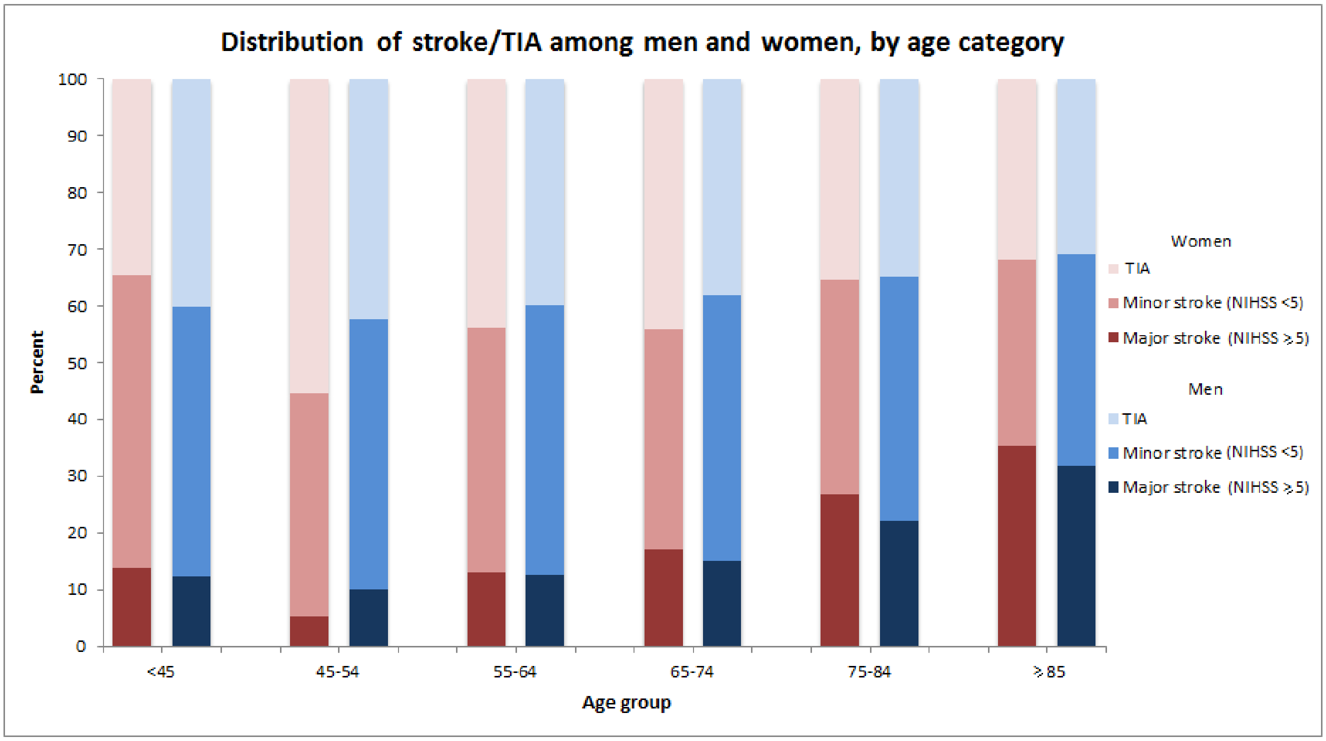

Figure I. Distribution of stroke/TIA among men and women, by age category

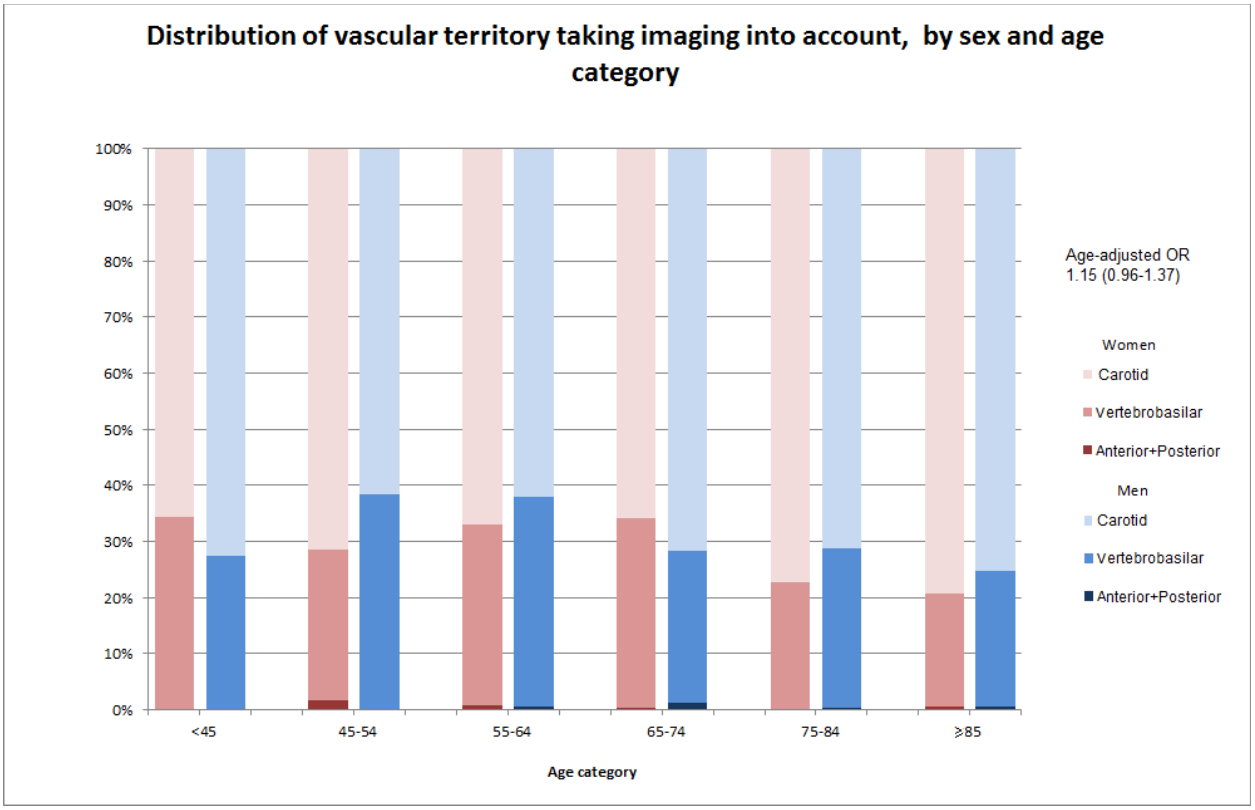

Figure II. Distribution of vascular territory taking imaging into account, by sex and age category

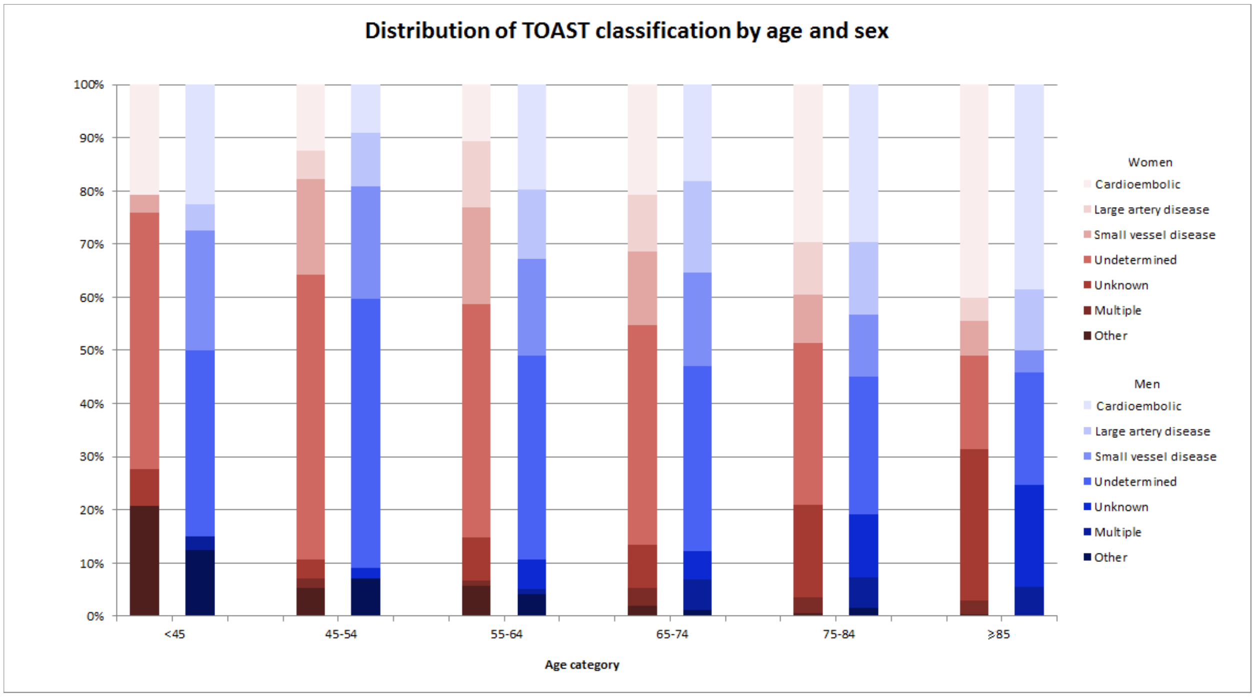

Figure III. Distribution of TOAST classification by age and sex

Results I. mRS scores 6 months following the ischemic stroke and change in mRS score from premorbid mRS to 6 months after ischemic stroke in women compared with men.

Women had a worse mRS score compared with men six month following the ischemic stroke, excluding patients with a TIA (crude OR 1.92; 95% CI 1.60-2.30 and age-adjusted OR 1.35; 95% CI 1.12-1.62). Results were very similar considering only individuals alive at 6 months (crude OR 1.84; 95% CI 1.50-2.27 and age-adjusted OR 1.47; 95% CI 1.19-1.81). Female sex was not associated with a higher risk of increased mRS score following stroke either in the whole cohort (age adjusted OR 1.04; 95% CI 0.85 - 1.27), or in the subgroup of patients aged 65 or older (age adjusted OR 1.01; 95% CI 0.81-1.26).
Results were unchanged when assessing mRS change as a binary outcome (age adjusted OR 1.00 (95% CI 0.80-1.26) and 0.93 (95% CI 0.73-1.20), respectively).
Among patients with a low premorbid mRS score (0-2), women were slightly more likely than men to have a score of three or more one month after the stroke (age adjusted OR 1.29; 95% CI 1.002-1.67).
